# Supplementary material for: Cryo-electron tomography reveals the binding and release states of the major adhesion complex from Mycoplasma genitalium
Source: PLoS Pathog. 2023 Nov 8;19(11):e1011761. doi: 10.1371/journal.ppat.1011761 (PMC10659161; doi:10.1371/journal.ppat.1011761)
Supplement: S1 Table — (DOCX) [file ppat.1011761.s001.docx]

**Supplementary Table 1: Refinement and validation statistics for the P110/P140 heterodimer**

| **Refinement** | **P140/P110**  **Dimer Class 1** | **P140/P110**  **Dimer Class 2** |
| --- | --- | --- |
| Initial Model used  (PDB code) | 6RUT and 6R3T  for the P110 stalk | P140/P110  Dimer Class 1 |
| Model resolution (Å) | 3.3 | 3.7 |
| FSC threshold | 0.143 | 0.143 |
| Model resolution range (Å) | 2.8-5.0 | 3.2-7.0 |
| Map sharpening B factor (Å^2^) | -113.8 | -108.7 |
| **Model composition** |  |  |
| Non-hydrogen atoms | 17017 | 17017 |
| Protein residues | 2187 | 2187 |
| Ligands | 0.00 | 0.00 |
| **B factors (Å^2^)** |  |  |
| Protein | 162.04 | 162.04 |
| Ligand | 0.00 | 0.00 |
| **R.m.s. deviations** |  |  |
| Bond length (Å) | 0.005 | 0.011 |
| Bond angles (°) | 0.591 | 0.942 |
| **Validation** |  |  |
| MolProbity score | 2.01 | 2.2 |
| Clashscore | 11.63 | 18 |
| Poor rotamers (%) | 0.26 | 0.42 |
| **Ramachandran plot** |  |  |
| Favored (%) | 93.43 | 93.16 |
| Allowed (%) | 6.52 | 6.80 |
| Disallowed (%) | 0.05 | 0.05 |
