# Supplementary material for: Cryo-electron tomography reveals the binding and release states of the major adhesion complex from Mycoplasma genitalium
Source: PLoS Pathog. 2023 Nov 8;19(11):e1011761. doi: 10.1371/journal.ppat.1011761 (PMC10659161; doi:10.1371/journal.ppat.1011761)
Supplement: S2 Table — (DOCX) [file ppat.1011761.s002.docx]

**Supplementary Table 2: Cryo-ET and cryo-EM data collection and processing parameters**

| **Data Collection** | **Cryo-ET Native nap particle** | **Cryo-EM Isolated nap particle** | **Cryo-EM Isolated**  **nap particle with 6**′-**SL** | **Cryo-EM**  **Heterodimer** |
| --- | --- | --- | --- | --- |
| Microscope | Titan Krios | Titan Krios | Titan Krios | Titan Krios |
| Detector | Gatan K2 Summit | Gatan K2 Summit | Gatan K3 Summit | Gatan K2 Summit |
| Acquisition Software | SerialEM 3.8 | SerialEM 3.8 | SerialEM 3.1.3 | SerialEM 3.8 |
| Magnification | 105,000 x | 130,000 x | 105,000 x | 130,000 x |
| Pixel size (Å) | 0.65 | 1.05 | 0.837 | 1.05 |
| Total electron dose (e^-^/Å^2^) | 120 | 50 | 48 | 50 |
| Dose rate (Å^2^/s^-1^) | 8 | 8 |  | 8 |
| Number of Frames | 10 | 34 | 50 | 34 |
| frame time (s) | 0.1 | 0.2 |  | 0.2 |
| Defocus range (µm) | -3.00 | -1 to -4 |  | -1 to -4 |
| Tomograms used | 420 | n/a | n/a | n/a |
| Micrographs used | n/a | 2,545 | 7,394 | 2,545 |
| **Processing** |  |  |  |  |
| Software | Artiatomi | cryoSPARC v3.3.2 | cryoSPARC  v4.2 | cryoSPARC  v3.3.2 |
| Motion correction | Artiatomi | cryoSPARC v3.3.2 | cryoSPARC  v4.2 | cryoSPARC  v3.3.2 |
| CTF estimation | Artiatomi | cryoSPARC v3.3.2 | cryoSPARC  v4.2 | cryoSPARC  v3.3.2 |
| Total extracted particles | 36,720 | 1,237,922 | 702,386 | 729,158 |
| After 2D classification | n/a | 216,630 | 76,926 | 290,179 |
| Number of refined particles | Average: 36,720  Open class: 9,689  Closed class:13,870 | Class 1: 18,408 | 50,318 | Class 1: 149,542 |
|  |  | Class 2: 31,616 |  | Class 2: 67091 |
| Symmetry | C2 | C2 | C1 | C1 |
| Map sharpening B factor (Å^2^) | / | Class 1: -578.4 | -402 | Class 1: -113.7 |
|  |  | Class 2: -377.9 |  | Class 2: -108.7 |
| Model resolution (Å) | Average: 11  Open class: 17  Closed class: 18 | Class 1: 8.33 | 7.4 | Class 1: 3.29 |
|  |  | Class 2: 7.34 |  | Class 2: 3.7 |
| FSC threshold | 0.143 | 0.143 | 0.143 | 0.143 |
