# Supplementary material for: Cryo-electron tomography reveals the binding and release states of the major adhesion complex from Mycoplasma genitalium
Source: PLoS Pathog. 2023 Nov 8;19(11):e1011761. doi: 10.1371/journal.ppat.1011761 (PMC10659161; doi:10.1371/journal.ppat.1011761)
Supplement: S3 Table — (DOCX) [file ppat.1011761.s003.docx]

**Supplementary Table 3: Cross-linked residues between P1 and P40/P90 [21] and the corresponding residues of P140 and P110 based on structural homology**

|  | *Mycoplasma pneumonia* | | *Mycoplasma genitalium* | |
| --- | --- | --- | --- | --- |
|  | **P1** | **P40/P90** | **P140** | **P110** |
| tight interface: | LYS 687 | LYS 268 | LYS 649 | LYS 230 |
|  | GLU 754 | LYS 905 | GLU 715 | THR 730 |
|  | LYS 751 | LYS 987 | PHE 711 | ILE 811 |
|  | LYS 769 | LYS 905 | LEU 729 | THR 730 |
| loose interface: | LYS 1289 | LYS 818 | VAL 1169 | LYS 643 |
|  | LYS 1376 | LYS 727 | LYS 1220 | ARG 553 |
|  | LYS 1376 | LYS 842 | LYS 1220 | SER 667 |
