# Supplementary figures and images for: Cryo-electron tomography reveals the binding and release states of the major adhesion complex from Mycoplasma genitalium
Source: PLoS Pathog. 2023 Nov 8;19(11):e1011761. doi: 10.1371/journal.ppat.1011761 (PMC10659161; doi:10.1371/journal.ppat.1011761)

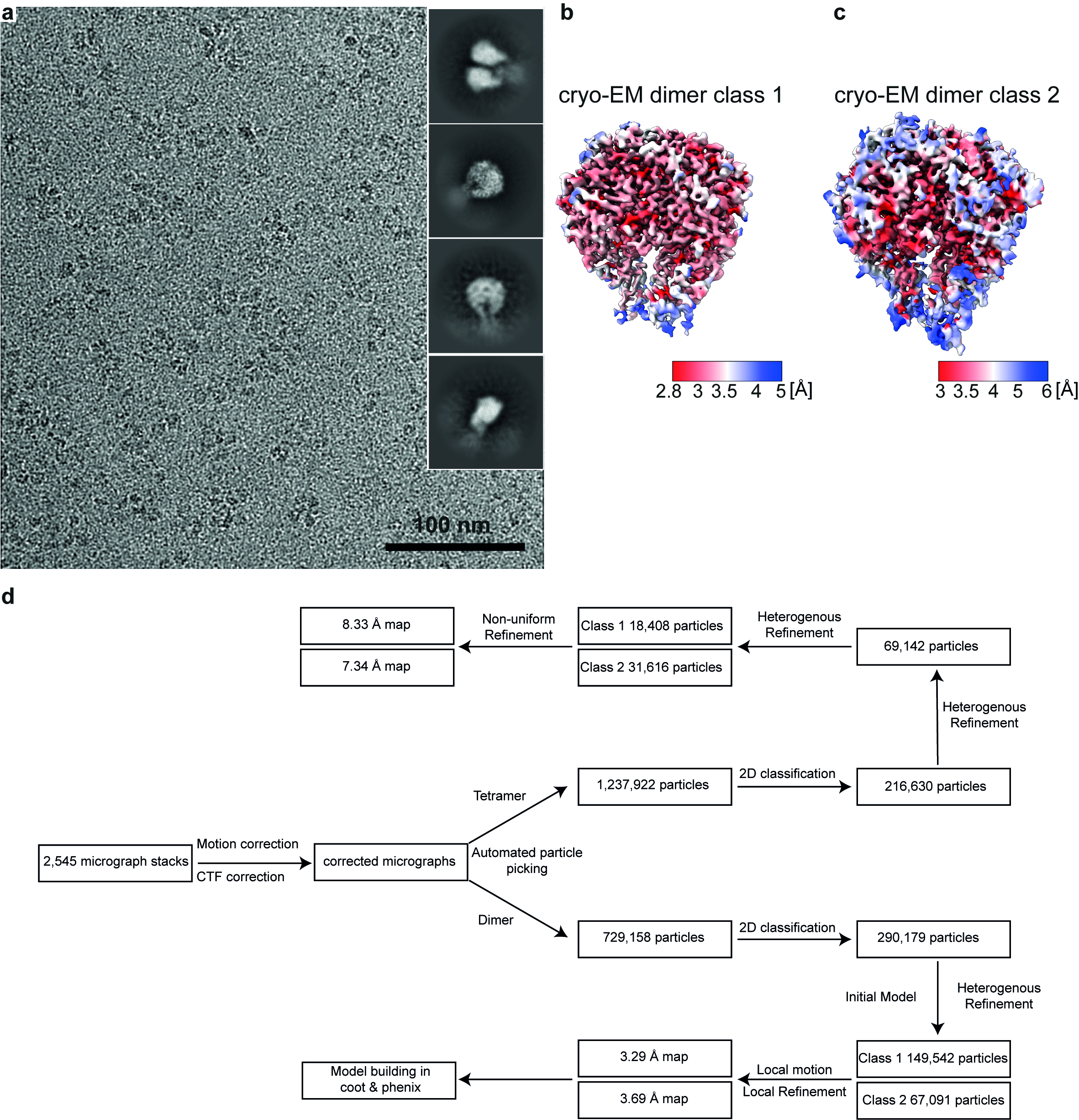

Supplement: S1 Fig — (a) Representative micrograph and 2D classes of the nap particle complex. Local resolution map of (b) cryo-EM dimer class 1 and (c) cryo-EM dimer class 2 illustrates the poorer local resolution of the stalks. (d) Schematic overview of the cryo-EM processing workflow to obtain the different nap particle classes. (TIF) [file ppat.1011761.s004.tif]

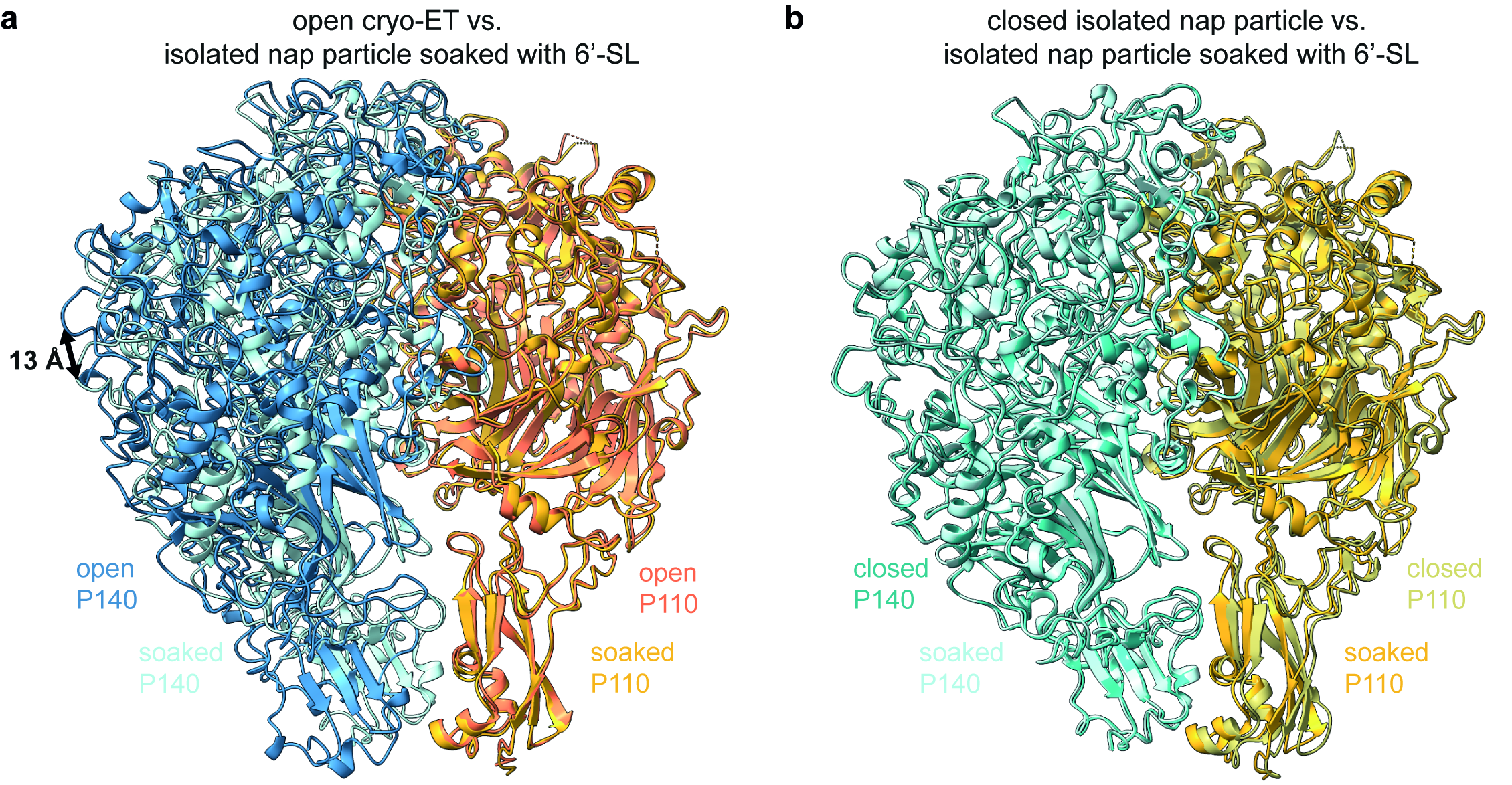

Supplement: S2 Fig — (a) Comparison of the MDFF of the 6′-SL-soaked nap particle complex with the MDFF of the ‘open’ state (8PC0) found in the cryo-ET data. Both MDFFs were performed in a similar manner using the identical starting models. Aligning the models on P110 shows, that the P140s are shifted by about 13 Å. Comparing the model of the 6′-SL-soaked nap particle with the (b) ‘closed’ model from the cryo-EM heterodimer (8PBX), no larger shift can be measured. It can be concluded that the 6′-SL-soaked nap particle resembles the ‘closed’ state. (TIF) [file ppat.1011761.s005.tif]

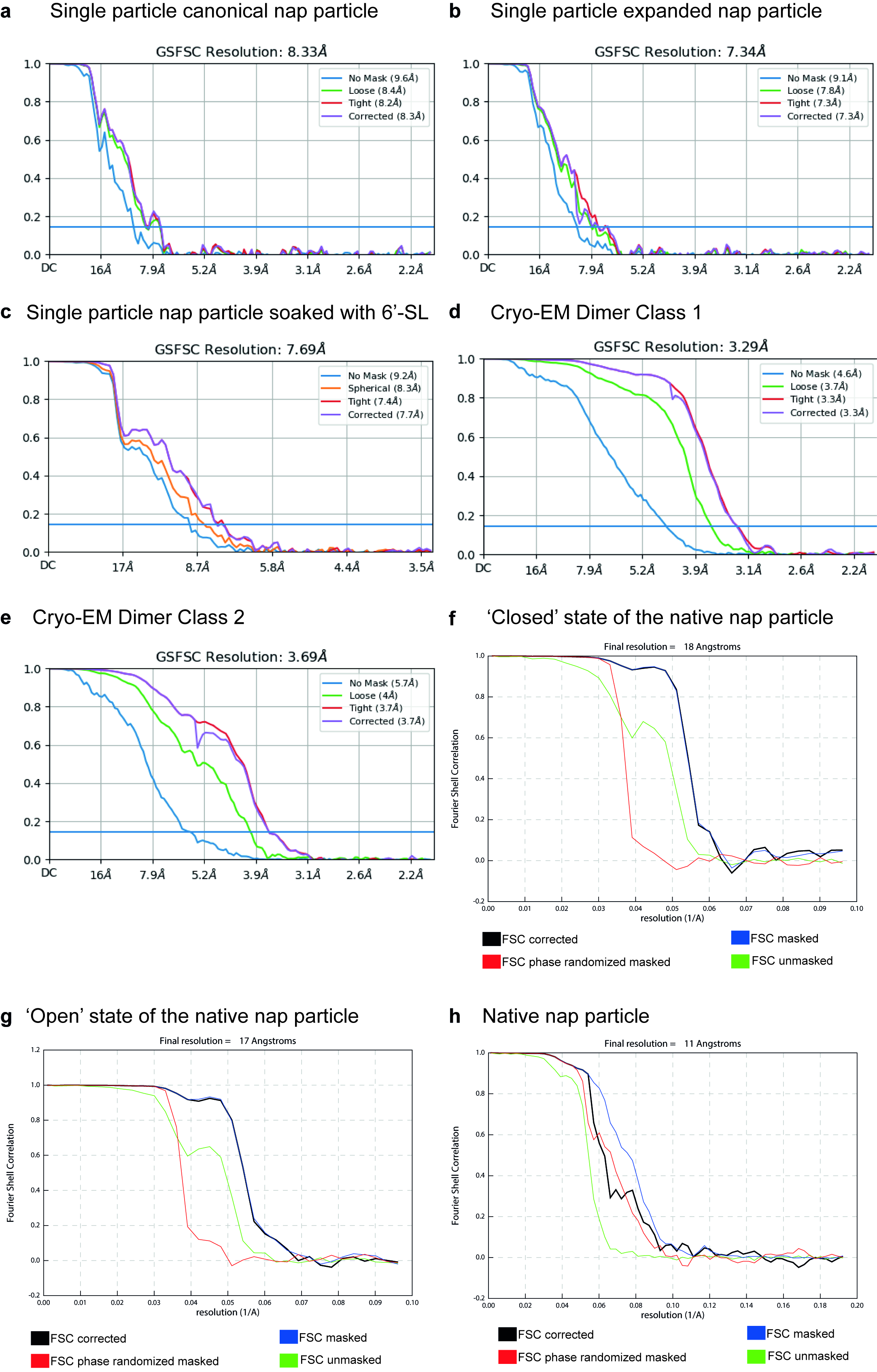

Supplement: S3 Fig — Fourier shell correlation of the (a) single particle canonical nap particle, (b) single particle expanded nap particle, (c) single particle nap particle soaked with 6′-SL, (d) cryo-EM dimer Class 1, (e) cryo-EM dimer Class 2, (f) ‘closed’ state of the native nap particles, (g) ‘open’ state of the native nap particles, (e) native nap particle. Resolution was calculated according to the gold standard criterion of FSC 0.143. (TIF) [file ppat.1011761.s006.tif]

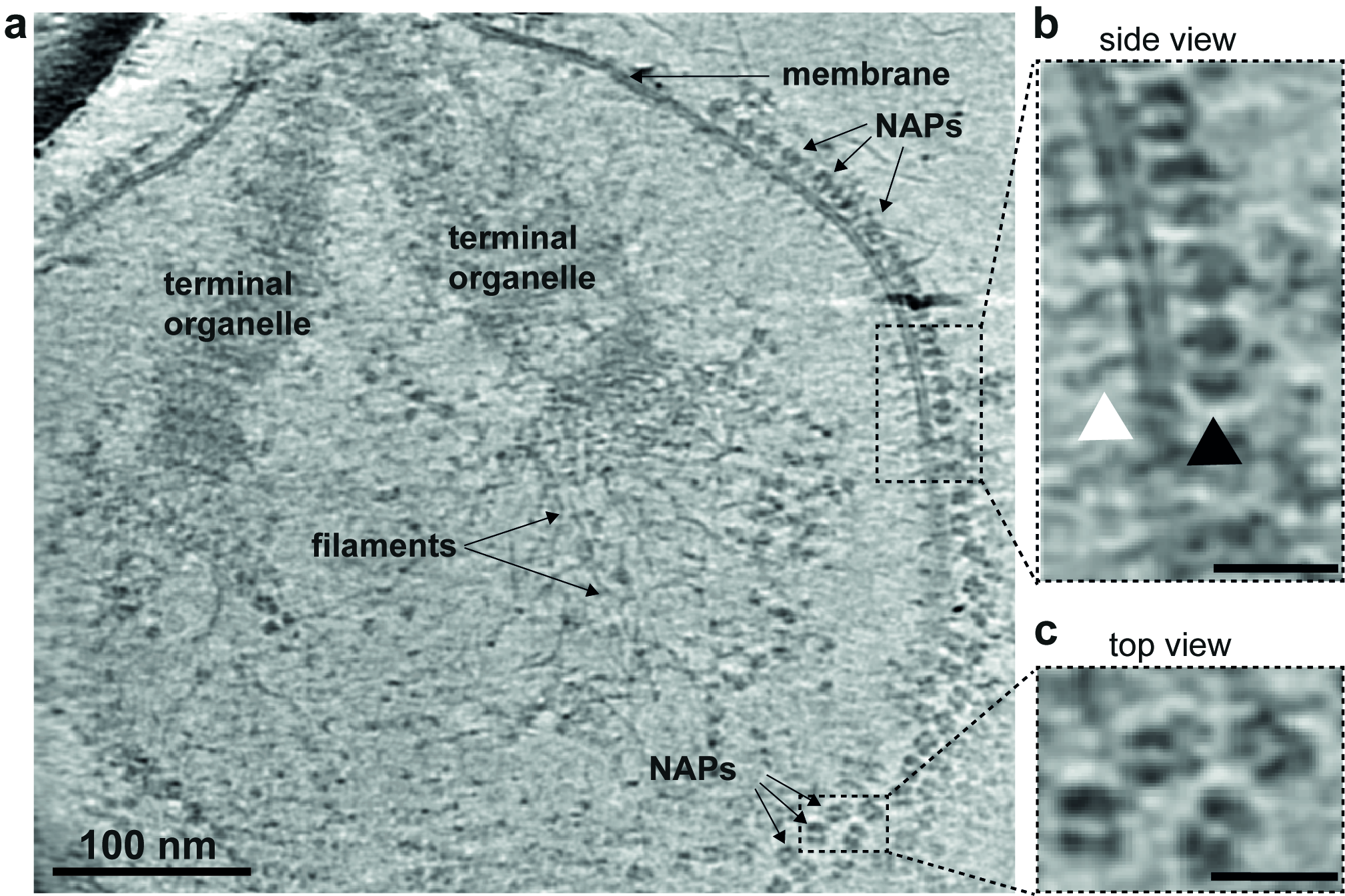

Supplement: S4 Fig — (a) Slice through a tomographic reconstruction of an M. genitalium ghost cell, which shows the intracellular terminal organelles, the surface exposed nap particles, the bacterial membrane and filaments attached to the terminal organelle. (b) Zoomed in region of nap particle complexes as a side view, showing the extracellular (black arrowhead) and intracellular region (white arrowhead) of the complex. (c) Zoomed in region of nap particle complexes as a top view. Scalebar of (b) and (c) is 20 nm. (TIF) [file ppat.1011761.s007.tif]

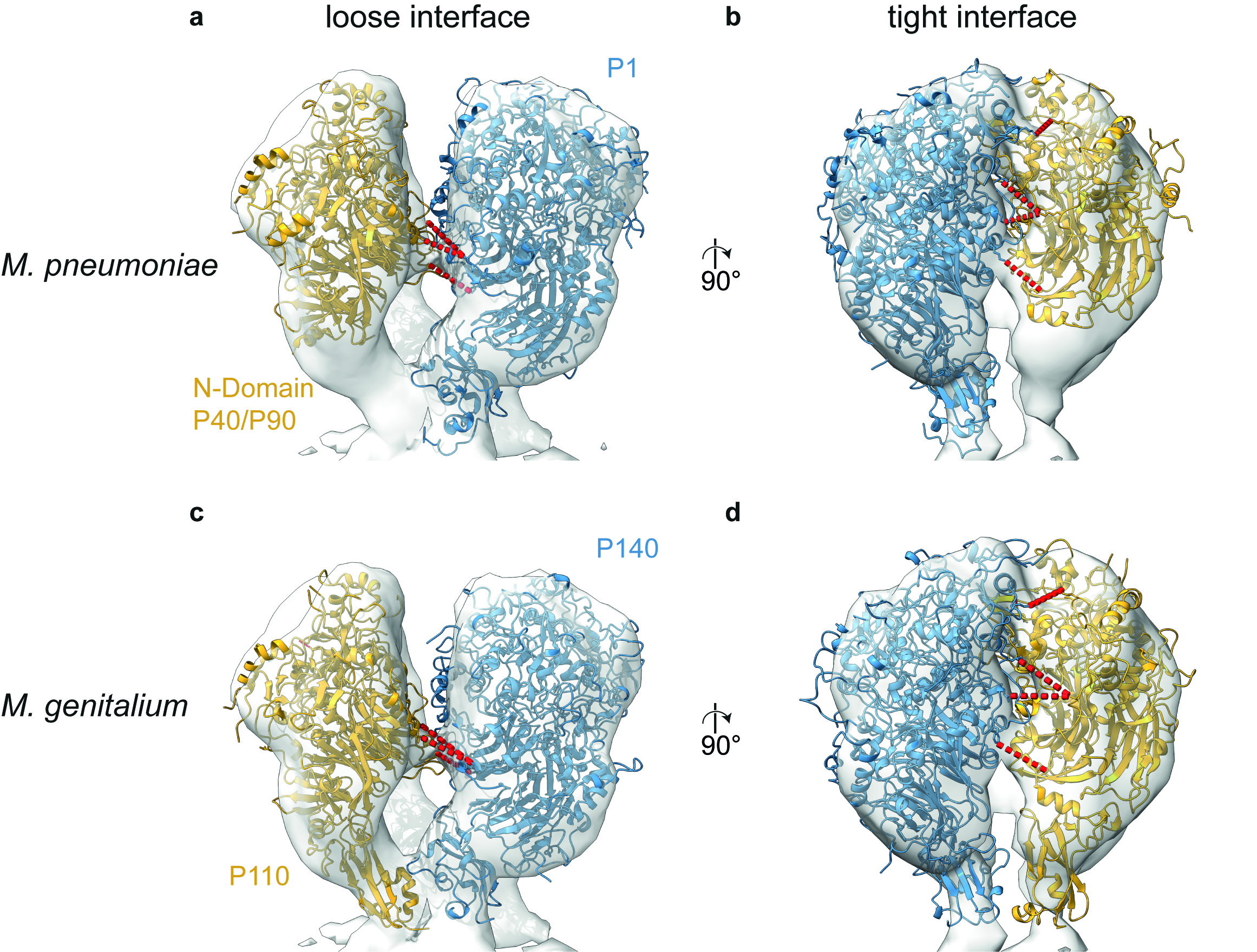

Supplement: S5 Fig — (a,b) For the M. pnuemoniae nap particle, the N-domain of P40/P90 (6RJ1) and P1 (6RC9) were fitted into the extracellular density of the cryo-ET nap particle from M. genitalium. Cross-linking data from M. pneumoniae 21 suggests multiple interactions (colored in red) of the nap particle complex within the (a) loose and (b) tight interface. (c,d) For the M. genitalium nap particle, P110 (6R3T) and P140 (6S3U) were fitted into the extracellular density of the M. genitalium cryo-ET nap particle. The interactions (colored in red) correspond to the M. pneumoniae residues at the (c) loose and (d) tight interface. P110 and the N-domain of P40/P90 are shown in yellow and P1 and P140 are shown in blue. (TIF) [file ppat.1011761.s008.tif]
