# Supplementary material for: Cryo-electron tomography reveals the binding and release states of the major adhesion complex from Mycoplasma genitalium
Source: PLoS Pathog. 2023 Nov 8;19(11):e1011761. doi: 10.1371/journal.ppat.1011761 (PMC10659161; doi:10.1371/journal.ppat.1011761)
Supplement: Attachment — Submitted filename: sprankelRebuttal_LS.docx [file ppat.1011761.s011.docx]

**Rebuttal letter: Sprankel et al. “Cryo-electron tomography reveals the binding and release states of the major adhesion complex from *Mycoplasma genitalium***

**”**

We would like to thank all reviewers and the editor for their encouraging comments and very constructive suggestions for our manuscript.

**Response to editorial comments:**

Three reviewers and I have evaluated the manuscript, and as you can see, there was considerable disagreement about whether the work represents a sufficiently great advance to warrant publication in PLOS Pathogens. I find that it potentially does, but there are some important matters to address concerning the description of the results and their interpretation. I agree with Reviewer 1 that although these static images could relate to the mechanism of motility, there isn't sufficient evidence to be quite as conclusive about that connection as written in the Discussion section. Overall, in addition to addressing the suggestions of the reviewers and my own comments below, the limitations of this work (including the absence of substrate in the structures) should be made explicitly clear, and how this work is a significant advance from other published work on both the M. genitalium and M. pneumoniae structures should be described.

The editor has requested to address some important matters concerning the description and interpretations of the results with respect to the conclusions reached in the paper regarding the relation of static images to the mechanism of motility. Therefore, we have adapted the discussion section and removed the last paragraph regarding conclusions reached about the mechanism of motility. Although adhesion and motility are inextricably linked, we now rather present the discussion in terms of only the conformational changes observed. We also add a paragraph in the discussion regarding the limitations and advances of this work compared to previous work.

Additionally, we now explicitly state the limitations of this work in the paper and state which structures contain substrate, and which do not.

**Limitations of the work:** Even though sialylated oligosaccharides are present in the native nap particle and the isolated nap particle was soaked with 6’SL, the resolution is insufficient to resolve the substrate in the binding site.

**Advances of the work:** Structures of the entire heterodimer at 3.3 A resolution, including an alternate P140 stalk conformation resolved at 3.7 A resolution. Structure of the entire native nap particle complex at 11 A resolution, including two distinct previously unresolved classes depicting the ‘open’ and ‘closed’ state in the cellular context. Structure of the previously unresolved intracellular region of the native complex at 11 A resolution, thereby providing a model of the entire nap particle. All novel structures are deposited in public repositories.

We added to the discussion: “The structure of the entire native nap particle by cryo-ET depicts the ‘open’ and ‘closed’ state in the cellular context, which is not the case for purified nap particles. The density of the previously unresolved intracellular region was solved at 11 Å resolution, thereby providing a model of the entire complex. Unfortunately, the resolution of the cryo-ET data is insufficient to visualize the sialylated oligosaccharides, however in the ‘open’ state, the distance between P110 and P140 at the tight interface is sufficiently large that the interfering loop no longer interferes with the β-hairpin of P110.

The word "nap" has been misused in recent manuscripts on the adherence complexes of Mycoplasma genitalium and Mycoplasma pneumoniae, and your next paper - whether it's this one or another - is a great opportunity to rectify the usage. "Nap" was never meant to stand in as the name of an adherence-associated complex of proteins. The word "nap," which was first used in regard to M. pneumoniae in 1982 (Hu et al., Science, 216:313-315), comes from the textile industry. It means "the raised hairs, threads, or similar small projections on the surface of fabric or suede" (from Oxford Languages). It is a collective noun, like "dirt." It refers to the appearance of the apparently raised material on the surface of the attachment organelle. Just like for dirt, we wouldn't call a single particle of dirt "a Dirt." We would call it "a dirt particle." If "P140/P110 complex" isn't sufficiently concise, I recommend calling this individual tetramer a "nap particle" - all lowercase, as proteins, chemicals, and other non-living biochemical entities are not capitalized in English.

We thank the editor for the suggestion regarding the word “nap” and have implemented the suggestion to change the wording to “nap particle” in all instances. We have thus also changed the title to read: “Cryo-electron tomography reveals the binding and release states of the major adhesion complex from *Mycoplasma genitalium*”.

**Response to reviewers comments (Major issues):**

**Response to reviewer #1:**

Reviewer #1: The manuscript by Sprankel et al focuses on Nap, a protein complex responsible for cytoadherence of Mycoplasma genitalium, and reveals the structural polymorphism of intact Nap on the cell surface by cryo-ET. Although the mechanism of Nap binding and release state with sialylated oligosaccharides is an important issue, this manuscript does not provide direct evidence that the ‘open’ and ‘closed’ conformation is involved in the mechanism. Moreover, the present manuscript represents more limited progress compared to the previously published paper on the structure of Nap published by the same laboratory. Extensive additional experiments are needed to draw convincing conclusion in this study.

1) The authors’ elucidation of the ‘open’ and ‘closed’ conformations of the Nap structure at a higher resolution than before is intriguing. However, direct evidence linking this structural variation to the function of Nap is lacking. The authors should demonstrate the structural difference in the presence of sialylated oligosaccharides, rather than in a solvent without the binding target. The present data lacks sufficient impact as the previous study that have already been reported on the comparisons between closed and open states. To support the authors’ assertion, it would be necessary to showcase the structural polymorphism of Nap during both binding and release state with sialylated oligosaccharides.
2) Although this manuscript demonstrates a large amount of data, the authors should be more cautious in their conclusions and avoid giving the impression that his work provides definitive evidence for binding and release state throughout the manuscript.
3) It is worth nothing that the observation of the structure of the intracellular regions is a notable achievement. However, no discernible differences were observed in this region between the open and closed states. Therefore, it is advisable to minimize the discussion regarding intracellular interactions.

We thank reviewer #1 for their comments. Reviewer #1 is concerned that this work does not provide direct evidence that the ‘open’ and ‘closed’ conformation is involved in the mechanism. Therefore, the reviewer requires us to demonstrate:

1) “the structural difference in the presence of sialylated oligosaccharides, rather than in a solvent without the binding target” and
2) that we “showcase the structural polymorphism of Nap during both binding and release state with sialylated oligosaccharides.”
3) That we “minimize the discussion regarding intracellular interactions”.

In response to the first concern, we have recorded additional large single-particle cryo-EM datasets of the nap particle soaked with 6’-SL. The ligand 6’-SL was added to the isolated nap particle before freezing and we then performed extensive classification, with the aim to capture different conformational states. Despite adding sialic acid to the purified protein, the open state could not be classified. Only the closed state was present. This provides evidence that a structured intracellular nap domain as found in the native nap particle *in situ* is required for coming to the open state. This is only the case in the cellular context. While in previous studies the “open state” was seen in tomograms -at a worse resolution than here- and the “closed” state has been reported for purified nap particles, here for the first time we demonstrate both states *in vivo*. In combination with the structure of the intracellular part and the high-resolution single particle data we have now a complete model of the “structural polymorphism of the nap particle”.

In response to the second concern, we now make it clear in the manuscript that our data of the native nap particle (*in-situ* tomography data) contains sialylated oligosaccharides as the cells are grown in complete medium where sialylated oligosaccharides are present. Although the resolution obtained until now is insufficient to resolve the ligand in the crown region, the fact that we obtain classes with the open conformation, which is the only conformation where the distance between P110 and P140 at the tight interface is sufficiently large so that the interfering loop no longer interferes with the β-hairpin of P110, is evidence that sialylated oligosaccharides may be bound to these nap particles depending on their state, even though the resolution is insufficient to resolve the substrate.

As it is common practice to infer molecular conformational change based on static states captured by structural biology techniques, we feel that our *in-situ* data of the entire nap particle in its native state in both open and closed conformation (not determined in our previous data) brings a substantial advance to the understanding of how conformational change occurs in the cellular context. Both reviewer #2 & #3 share this opinion, with reviewer #3 praising the results achieved from our tomographic data.

As we know that heterodimers cannot bind sialic acid (surface plasmon resonance, Aparicio et al. 2020, Supplementary Figure 5), we are sure that at least the tetrameric conformation is required to generate an open state. However, as we never see the open state in isolated nap particles, even though we soaked the isolated nap particle with 6′-SL , we infer that an intact intracellular part is required to achieve the open state of the heterodimer, which we only see in the native nap particle. This is our conclusion based on the beautiful *in-situ* data provided, which is also supported by the overwhelmingly enthusiastic responses of two of the reviewers.

We have changed the manuscript in order that the reader can understand that the native nap particle in its cellular context has sialylated oligosaccharides present. We added the sentence “Importantly, the mycoplasma cells are grown in complete medium where sialylated oligosaccharides are present.”

We thank the reviewer for the positive comment regarding the elucidation of the intracellular part as a notable achievement. In order to address the third concern regarding discussion on intracellular binding partners, we have removed all speculative discussion and only left relevant cross-linking for the P110 and P140 analogues.

Overall modifications of the manuscript to address reviewers #1 concerns:

We have added these sentences to the first paragraph and changed the title of the paragraph to: “Classification of the isolated nap particle complex shows a closed tight interface and the loose interface in different conformations”

“To structurally characterize the nap particle complex, we performed single particle analysis of two preparations: (i) the isolated nap particle complex directly purified from *M. genitalium* and (ii) the isolated nap particle complex soaked with 6′-sialyl-lactose (6′-SL) after purification (Material and Methods & Supplementary Figure 2). We obtained two datasets with 216,630 and 76,926 particles for each preparation, but only selected ~32 % and ~65 % of the particles that represented tetramers, respectively. Consistently for both preparations, the tight interface between P110 and P140 remains in the 'closed' state, where P140 occludes the sialic acid binding pocket of P110 (Figure 1 c & d, asterisk, Supplementary Figure 2). Thus, despite having sialylated oligosaccharides in the original medium and soaking the purified protein with 6′-SL, an open state of the tight interface, as observed in tomographic data^19^, cannot be seen,”

In the paragraph “Classification of the native nap particle from mycoplasma cells shows the tight interface in both the ‘open’ and the ‘closed’ state” we added:

“Although the resolution obtained is insufficient to resolve the sialylated oligosaccharides in the crown region, the fact that we obtain classes with the ‘open’ conformation is conclusive evidence for the bound or ready-to-bind state, since it is the only conformation with spacing sufficient to accommodate the ligand. Thereby, for the ‘open’ state of the nap particle (8PC0, EMD-17592), the crystal structure of P110 bound to 6’-sialyl-lactose (6’-SL;6R43) as well as the crystal structure of P140 (6RUT) were rigid-body fitted into the density map of the native nap particle.”

We have added this sentence to the paragraph “The P140 stalks adopt different conformations but do not suffice to change the crown arrangement to an open conformation”:

“This agrees with previous surface plasmon resonance experiments, which showed that sialylated compounds 3’-SL and 6’-SL (which bind to P110 alone) do not bind to the P140-P110 heterodimer in solution ^19^.”

**Response to reviewer #2:**

Reviewer #2: I read this body of work with interest. While am no expert in structural biology it is my opinion this is a rigorous piece of work that sheds new light on the mechanism of NAP function. I could not find any major drawbacks in experimental design.

We cordially thank reviewer #2 for their very positive comments.

**Response to reviewer #3:**

Reviewer #3: In their manuscript „Cryo-electron tomography reveals the binding and release states of the native Nap adhesion complex“, the authors Sprankel, Scheffer, Ermel & Frangakis report new insights into the major adhesion complex of Mycoplasma genitalium, the Nap complex.
My thoughts after reading the manuscript, briefly summarised:
It has been a long way from the first reports in 1984 about the special flask-shaped morphology of some motile Mycoplasmas (including M. genitalium) exhibited some undefined nap structures on the bacterial surface, to the deciphering of the amino acid sequence of adherence-mediating sites of the MgPa protein in 1992, to elucidating the transcriptonal linkage of P140 and P110 in 2003, and ending now in the description of the tetrameric conformation of the nap structure in its native state at an nice 11 Ångström resolution. What a nice piece of progress over time, and this manuscript definitively is part of the journey …

The authors once again proved the many interactions between P140 (also once called MgPa or MgpB or MG191) and P110 (or MgpC or MG192), forming the heterodimers, and how they together create the pocket for binding sialic acid receptors, making it obvious why these gene products are transcriptionally linked. In addition, the authors proved again the importance of studying molecules in its native state when one considers their now presented findings about the four-helices bundles of the intracellular regions of P140/P110, and that the tetrameric organization as well as the intracellular domains (and eventually other cytoplasmic proteins) are required for the correct conformation of the extracellular globular domains and the receptor binding site….. and that a “small difference in the distance between the stalk domains translates into a large difference in the crown region“ and thereby explaining how a small, distinct intracellular impetus may have a big effect at the extracellular binding site.
For me, this manuscript is clearly worth publishing.

We cordially thank the reviewer for their positive evaluation of our work and for stating so clearly the advances made by this work.

**Response to reviewers’ comments:**

We thank the reviewers and editor for the careful reading of the text and for their suggestions.

• 4 times a comma is missing, either upstream or downstream of “respectively“, pls also check commas when starting with “Subsequently“ or “Initially“

We thank the reviewer for this comment and have added commas accordingly.

• Missing blanks at “Figure 1 a&c“/“Figure 1 g,h“..(no &?)/“Figure 2g“/“Figure 2h“

We thank the reviewer for this comment and have checked all figure references in the text for consistency.

• lys1289 and lys818 should read Lys1289 and Lys818

We thank the reviewer for noticing this and have changed lys1289 and lys818 to Lys1289 and Lys818.

• “the pneumoniae cluster“ should read “the pneumoniae cluster“ (no species->no italics)

We thank the reviewer and have changed the text accordingly.

• “..inhibit adhesion (Vizarraga 2020)“ should read “..inhibit adhesion (26).“

We thank the reviewer for noticing this and have changed the references accordingly. The reference numbering has been updated.

• “CO2“ should read “CO2“

We thank the reviewer and have changed “CO^2^” to “CO2”.

• “Großlöblichau“ should read “Großlöbichau“

We thank the reviewer and have changed “Großlöblichau” to “Großlöbichau”.

• “Clearly misaligned particles…were removed for further processing“: really? Or were they instead excluded from further processing?

We thank the reviewer and have changed the text to read “Clearly misaligned particles were classified as bad particles and manually excluded from further processing.”

• “…box size of 300 pixel“ should read “…box size of 300 pixels“

We thank the reviewer and have changed “…box size of 300 pixel” to “…box size of 300 pixels”

• First line page 6 – change “are slightly wider apart” to “is slightly greater”

We thank the editor and have changed “are slightly wider apart” to “is slightly greater” in the text.

• Next to last paragraph of discussion – P30 is M. pneumoniae; P32 is M. genitalium

We thank the editor for this comment and acknowledge the ambiguity of this paragraph in applying the cross-linking data of *M.pneumoniae* to *M.genitalium*. We have adjusted the paragraph for a clearer understanding, and it now reads:

“Although crosslinking mass spectrometry of the *M. pneumonia* analogue for P110 shows five possible self-crosslinks and another ten cross-links with P140 in the intracellular regions, the intracellular region of P110 is not shown to interact with any other cytoskeleton proteins. Cross-linking mass spectrometry of the *M. pneumonia* analogue for P140, on the other hand, shows that it has two self-crosslinks and two additional crosslinks with the cytoskeleton proteins HMW1 and P32 (P30 in *M. pneumonia*), and is therefore likely responsible for signal transduction towards the rest of the motility machinery ^24^.”

• Make sure you've written "M. pneumoniae" and not "M. pneumonia" in all instances

We thank the editor and have changed all instances of " *pneumonia*" to "*pneumoniae*".

In the Discussion section, authors mention that “Evidence for this is the fact that mycoplasma cells with a decreased density of Naps on the cell surface show faster gliding velocity…” Please provide the appropriate reference supporting this statement.

This paragraph has been removed in compliance with the comments of the reviewer about the conclusion of our results on the motility.

This manuscript lacks the appropriate references to other related papers.

We thank the reviewer for pointing this out and would appreciate if they could please specify which references to add, and we would gladly comply.
